# Polymorphisms and Mutational Covariation Associated with Death in a Prospective Cohort of HIV/AIDS Patients Receiving Long-Term ART in China

**DOI:** 10.1371/journal.pone.0170139

**Published:** 2017-01-18

**Authors:** Pengtao Liu, Yi Feng, Jianjun Wu, Suian Tian, Bin Su, Zhe Wang, Lingjie Liao, Hui Xing, Yinghui You, Yiming Shao, Yuhua Ruan

**Affiliations:** 1 State Key Laboratory of Infectious Disease Prevention and Control, National Center for AIDS/STD Control and Prevention, Chinese Center for Disease Control and Prevention, Collaborative Innovation Center for Diagnosis and Treatment of Infectious Diseases, Beijing, China; 2 Anhui Center for Disease Control and Prevention, Hefei, Anhui Province, China; 3 Henan Center for Disease Control and Prevention, Zhengzhou, Henan Province, China; 4 Weifang Medical University, Weifang, Shandong Province, China; Istituto di Genetica Molecolare, ITALY

## Abstract

**Background:**

HIV drug resistance is associated with faster clinical progression of AIDS. However, the effect of significant polymorphisms and mutational covariation on mortality among HIV/AIDS patients receiving long-term antiretroviral therapy (ART), have rarely been studied.

**Methods:**

In this prospective cohort study from December 2003 to December 2014, we present a new computational modelling approach based on bioinformatics-based models and several statistical methods to elucidate the molecular mechanisms involved in the acquisition of polymorphisms and mutations on death in HIV/AIDS patients receiving long-term ART in China.

**Results:**

This study involved 654 ART-treated patients, who had been followed for 5473.4 person-years, a median of 9.8 years, and 178 died (25.2%, 3.3/100 person-years). The first regimens included AZT/d4T + NVP+ ddI (78.9%) or AZT/d4T + NVP+ 3TC (20.0%). We calculated an individual Ka/Ks value for each specific amino acid mutation. Result showed that 20 polymorphisms (E6D, Q18H, E35D, S37N, T39A, K43E, S68N, L74I, I93L, K103N, V106A, E169D, Y181C, G190A, Q197K, T200V, T200E, T215I, E224D and P225H) were strongly associated with AIDS related deaths. Among them, 7 polymorphisms (L74I, K103N, V106A, Y181C, G190A, T215I and P225H) were known to be drug resistance mutations, 7 polymorphisms (E6D, E35D, S37N, I93L, E169D, T200V and T200E were considered to be potential drug resistance mutations, and 6 polymorphisms (T39A, K43E, S68N, Q197K, T200V and E224D) were newly found to have an association with drug resistance mutations, which formed a complex network of relationships.

**Conclusions:**

Some polymorphisms and mutational covariation may be the important influencing factors in the failure of treatment. Understanding these mechanisms is essential for the development of new therapies, designing optimal drug combinations, and determining effective clinical management of individual patients.

## Introduction

China’s National Free Antiretroviral Treatment Program (NFATP) was initiated in 2003 and 471,140 patients have received ART by the end of 2015 [1]. NFATP is a community-based public health approach that has greatly reduced the mortality and morbidity caused by HIV infection [2–5]. However, many HIV-infected patients receiving antiretroviral therapy (ART) have experienced treatment failure, increasing the risk of HIV-related deaths. The emergence of drug resistance variants is the main obstacle to the effectiveness of ART. HIV can evolve drug resistance rapidly in response to new drug treatments, often, through a combination of multiple mutations [6–8]. For most of antiretroviral (ARV) drugs, a single resistance mutation does not result in maximal resistance [9]. HIV continually accumulates mutations in its genome, and some of these mutations allow HIV to become resistant to individual ARV drugs.

The analyses of databases containing HIV-1 sequences from drug-naive and ART-treated patients have revealed the emergence of these accessory mutations could be related to the evolutionary pathway leading to the selection of drug-resistant isolates [10–12]. The selection pressure-based method is a useful way to explore the critical sites of mutations. In addition, this method can effectively detect the actual drug-resistance and mutational covariation [13]. HIV-1 functional constraints are usually expected to limit the amino acid substitution rates, resulting in a higher conservation of functional sites on the rest of the protein surface. Jaccard similarity coefficient, an extremely specific measure of covariation [14], was used to determine the covariation between mutations.

The HIV-1 subtype B' (Thai-B) plays unique roles in the genesis of the HIV-1 epidemic in China [15]. It is indicated that HIV-1 subtype B' is a single founding strain responsible for HIV-1 outbreaks among former plasma donors (FPDs) in China. However, the drug resistance of HIV-1 subtype B' has rarely been reported. The evolution of resistance mutation under ART, especially its relation to long-term ART (a median of 9.8 years) effect needs further investigation.

This study sought to determine whether certain mutational patterns were more prevalent in patients dead from HIV/AIDS compared to those alive. Hypothesis testing and selection pressure-based method were performed to determine the polymorphisms and mutations between deceased and living patients.

## Materials and Methods

### Study Population

This observational cohort study enrolled patients in Henan Province and in Anhui Province, both in China, from December 2003 to December 2004. They were the first groups of patients to receive the free ART drugs in China. Patients who were more than 18 years old, started ART between 2003 and 2004, and were willing to provide informed consent eligible for enrolment.

Data on demographics and risk factors for HIV infection were collected at baseline. Patients were followed up every six months up to 31 May 2010, or to stopping ART, death or loss to follow-up, and then every 12 months until December 2014. The information on treatment and outcomes was collected at every follow-up visit, including last combination antiretroviral regimen, missed ARV drugs within a month, CD4 T cell count (per μL) and viral load (copies/mL).

This observational cohort study was approved by the Institutional Review Board of the National Center for AIDS/STD Control and Prevention, Chinese Center for Disease Control and Prevention. Written informed consent was obtained from all of the subjects at the time of data collection.

### Laboratory Tests

Blood samples were collected in ART clinics and processed in the CDC laboratories. The CD4 T cell count was measured within 24 hours after sampling by flow cytometry. The laboratory of NCAIDS (National Center for AIDS/STD Control and Prevention) in Beijing performed the tests for HIV viral load and sequences. Plasma HIV RNA was quantified with real-time NASBA (NucliSense Easy Q, bioMerieur, France) or COBAS (Roche Applied Science, Germany). HIVDR (HIV drug resistence) genotyping was carried out by an in-house PCR protocol for samples at baseline or follow-up with viral loads of more than 1000 copies/mL. The resulting fragment of the HIV pol gene (full protease amino acids from 1 to 99; part of the RT (reverse transcriptase) amino acids from 1 to 255) was amplified, purified, and bidirectionally sequenced using an ABI3100 sequencer (Applied Biosystems, Foster City, CA, U.S.A.) [16, 17]. For individuals from whom multiple sequences were available, the last sequence of therapy was used in the analyses. Sample pol gene sequences were compared to a consensus sequence using HIVdb software (Stanford HIV Drug Resistance Database) to detect drug resistance mutations. We involved all drug resistance mutations that conferred low, intermediate or high-level resistance.

### Data Processing and Statistical Analysis

Data were double-blinded, entered, and compared using EpiData software (EpiData 3.1 for Windows; The EpiData Association Odense, Denmark). Questionnaire and laboratory data were analyzed using Statistical Analysis System (SAS 9.3 for Windows; SAS Institute Inc., NC, U.S.A.). Mutation rates were computed and tested for significance based on chi-square and Fisher’s exact tests. P-values < 0.05 were considered to be statistically significant, and all tests of significance were two-sided.

### Phylogenetic Analyses

PhyML 3.0 was used to construct a maximum likelihood phylogenetic tree with all of the sequences obtained. Tree topologies were heuristically searched using the subtree pruning and regrafting procedure [18]. The branch significance was analyzed by bootstrap with 1000 replicates and inter-subject distances were calculated. The final tree was viewed using MEGA5.0 software and FigTree v1.3.1 (http://beast.bio.ed.ac.uk), as previously described [19].

### Ka/Ks Computation

The selective pressure on a protein-coding gene was measured by comparing silent (synonymous) and replacement (nonsynonymous) substitution rates, often referred to as *Ka*/*Ks* (amino acid mutations over synonymous mutations) or *dn/ds* (nonsynonymous mutations over synonymous mutations) [20]. A higher replacement than silent rates provides unequivocal evidence for adaptive evolution driven by Darwinian selection. Since HIV has a high transition-to-transversion ratio, we calculated an individual *Ka*/*Ks* value for each specific amino acid mutation, instead of calculating *Ka*/*Ks* for an individual gene or codon [13]. *Ka/Ks* is calculated using the following formula developed by Li *et al*. [21]:

$$\frac{K_{a}}{K_{s}}=\frac{N_{a}/N_{s}}{\left(n_{a,t}f_{t}+n_{a,v}f_{v})/(n_{s,t}f_{t}+n_{s,v}f_{v}\right)}$$

where *N*_*a*_ and *N*_*s*_ are the numbers of non-synonymous mutations and synonymous mutations observed at the codon, respectively; *n*_*a*,*t*_ is the number of possible transition mutations that will change the amino acid; *n*_*s*,*t*_ is the number of possible transition mutations that are synonymous; *n*_*a*,*v*_ and *n*_*s*,*v*_ are the equivalent numbers for transversions; and *f*_*t*_ and *f*_*v*_ are the transition and transversion frequencies, respectively.

If an amino acid change is neutral, it will be fixed at the same rate as a synonymous mutation, with *Ka/Ks* = 1. If the amino acid change is deleterious, however, purifying selection will reduce its fixation rate so that *Ka/Ks* < 1. *K*a/*K*s values significantly lower than 1 are regarded as undergoing purifying selection and therefore, may have a functionally or structurally important role. When the amino acid change offers a selective advantage, it will be fixed at a higher rate than a synonymous mutation, with *Ka/Ks* > 1. *K*a/*K*s values significantly greater than 1 are indicative of positive Darwinian selection, suggesting adaptive evolution [20]. *Ka*/*Ks* values are calculated for amino acid sites 1 to 99 in PR and 1 to 255 in RT.

A LOD confidence score is calculated for a mutation to be under a positive selection pressure according to the following formula [13]:

LOD=−log10⁡∑i=NYaXaN(Ni)qi(1−q)N−i

$$q=\frac{n_{a,t}f_{t}+n_{a,v}f_{v}}{3f_{t}+6f_{v}}$$

where *N* is the total number of mutations observed in the codon. If positive selection (*Ka*/*Ks* > 1) has LOD scores of 2 or greater, then the positive selection is significant.

An individual *Ka/Ks* value for each specific amino acid mutation was calculated. Then the association of these mutations with the drug resistance was investigated based on the criteria: (1) *Ka*/*Ks* > 1, LOD > 2; (2) Frequency of mutations in deceased patients was significantly larger than that in the living patients; (3) the non-synonymous mutations with low frequency (<1% deceased patients) were excluded.

### Jaccard Similarity Coefficient

For a pair of mutations X and Y, Jaccard similarity coefficient is calculated as:

J=NXY(NXY+NX0+N0Y)

where N_XY_ represents the number of sequences containing mutation X and Y; N_X0_ represents the number of sequences containing X, but not Y; and N_0Y_ represents the sequences containing Y, but not X [14].

Jaccard similarity coefficient uses only those sequences in which at least one of a pair of mutations is present. To test whether the observed Jaccard similarity coefficients are statistically significant, the Fisher exact test is used (2,000 replicates used in the Monte Carlo test). Jaccard similarity coefficient is calculated as the mean Jaccard similarity coefficient after 2,000 random rearrangements of the X or Y vector (containing 0 or 1 for presence or absence of a mutation, respectively) [14]. If the OR (odds ratio) > 1, the mutation pair is deemed to be positively correlated, and it is considered to be negatively correlated if the OR < 1. The software, Cytoscape, is then used to construct the relationship between these mutations [22].

## Results

### Study Population Characteristics

Of 691 study participants, 37 patients were excluded from this study, including 30 who stopped ART for a long time, and 7 patients whose deaths were not related with AIDS (3 patients committed suicide, 3 patients died in accidents and 2 patients died of a brain hemorrhage). This study involved 654 ART-treated patients, who had been followed for 5473.4 person-years, a median of 9.8 years, and 178 died (25.2%, 3.3/100 person-years) before the last follow-up in 2014. A majority of individuals were women (57.2%); young and middle-aged adults (25–44 years old) at enrolment accounted for 74.8%; only 2.8% had received post-secondary school education; most of them finished secondary school or less; 84.1% married and living with partner. Blood transfusion was the major route of transmission, accounting for 93.6% of all cases. The first regimens included AZT/d4T + NVP+ ddI (78.9%) or AZT/d4T + NVP+ 3TC (20.0%). The last regimens included AZT/d4T + NVP+ ddI (17.7%) or AZT/d4T + NVP+ 3TC (25.4%) or TDF+LPV+3TC (52.5%). Most patients were good in medication adherence, and only 6.4% missed ARV drugs within a month mainly because of working outside. 15.8% of patients initiated ART with a CD4 T cell count of higher than 500 cells/mL, 19.7% with a CD4 T cell count of between 350 and 499 cells/mL, 29.4% with a CD4 T cell count of between 200 and 349 cells/mL, and only 35.2% had a CD4 T cell count of less than 200 cells/mL. All the patients in the study were identified to be infected by HIV-1 subtype B' as determined by Neighbor-joining genetic analysis of pol sequences of the viruses obtained from plasma samples of the HIV-1-infected patients using PCR technique (Table 1).

**Table 1:** Baseline characteristics of HIV patients in the study cohort.

	Total Number (%)
**Total number**	654
**Years of ART (Median)**	5473.4(9.8)
**Death (/100 person-year)**	178(3.3)
**Gender**	
**Male**	280(42.8)
**Female**	374(57.2)
**Age at inclusion (years)**	
**25–44**	489(74.8)
**45–60**	149(22.8)
**60–71**	16(2.5)
**Education**	
**No schooling**	198(30.3)
**Primary school**	283(43.3)
**Secondary school**	147(22.5)
**Post-secondary school**	18(2.8)
**Missing data**	8(1.2)
**Marital status**	
**Married and living with partner**	550(84.1)
**Other**	104(15.9)
**Route of HIV infection**	
**Blood transfusion**	612(93.6)
**Plasma transfusion**	12(1.8)
**Sexual intercourse**	20(3.1)
**Other or unknown**	8(1.2)
**First combination antiretroviral regimen**	
** AZT/d4T NVP ddI**	516(78.9)
** AZT/d4T NVP 3TC**	131(20.0)
** Other**	7(1.1)
**Last combination antiretroviral regimen**	
** AZT/d4T NVP ddI**	116(17.7)
** AZT/d4T NVP 3TC**	166(25.4)
**3TC TDF LPV**	343(52.5)
**Other**	29(4.4)
**Missed ARV drugs within a month**	
** No**	611(93.6)
** Yes**	42(6.4)
**CD4 T cell count (per μL)**	
**<200**	230(35.2)
**200–349**	192(29.4)
**350–499**	129(19.7)
**≥500**	103(15.8)
**Viral load (copies/mL)**	
**<1000**	240(36.7)
**1000–9999**	55(8.4)
**10000–99999**	133(20.3)
**100000–999999**	190(29.1)
**≥1000000**	36(5.5)
**Subtype**	
**B'**	654(100.0)
**other**	0(0.0)

### Hypothesis Testing and Selection Pressure Computation for Individual Mutations

Mutations in the RT and PR of HIV-1 viruses from 178 patients who had died of AIDS and the 476 who were still living were analyzed. We used chi-square and Fisher’s exact tests (when sample sizes were small) to determine the critical mutations in the deceased group, and 20 death-associated polymorphisms were identified. These polymorphisms include E6D, Q18H, E35D, S37N, T39A, K43E, S68N, L74I, I93L, K103N, V106A, E169D, Y181C, G190A, Q197K, T200V, T200E, T215I, E224D and P225H. Notably, 7 (35%) mutations (L74I, K103N, V106A, Y181C, G190A, T215I and P225H) among those classified were known to be associated with drug resistance according to the HIVdb Program in the Stanford HIV Drug Resistance Database. Most drug resistance mutations (L74I, K103N, Y181C, and G190A) had a high frequency in two groups. They were all positive Darwin selection mutations (*Ka*/*Ks* > 1, LOD > 2), except for V106A and P225H. Others 13 (65%) (E6D, Q18H, E35D, S37N, T39A, K43E, S68N, I93L, E169D, Q197K, T200V, T200E and E224D) had not been previously reported to be drug resistance mutations. Among these newly found mutations, 7 mutations (E6D, E35D, S37N, I93L, E169D, T200V, and T200E) were considered to be potential drug resistance mutations (Table 2).

**Table 2:** The changes of significant polymorphisms between deceased and living patients.

Mutation^a^	Deceased Patients				Living Patients			OR	p^b^
	Frequency(n = 178) Ka/Ks		LOD	Region	Frequency(n = 476) Ka/Ks		LOD		
**Q18H**	1.7%(3)	2.6	2.0	PR	0%(0)	_	_	Inf^c^	0.02
**E35D**	68%(121)	518.4	143.2	PR	44.5%(212)	929.6	253.9	1.5	<0.01
**S37N**	87.6%(156)	156.0	73.4	PR	56.5%(269)	269.0	127.7	1.6	<0.01
**I93L**	84.8%(151)	1444.8	224.5	PR	53.2%(253)	2471.9	Inf	1.6	<0.01
**E6D**	14%(25)	19.9	19.5	RT	4.8%(23)	18.9	17.4	3.2	<0.01
**T39A**	1.7%(3)	3.9	1.3	RT	6.1%(29)	37.8	13.9	0.3	0.02
**K43E**	4.5%(8)	1.1	1.5	RT	0%(0)	_	_	Inf	<0.01
**S68N**	1.7%(3)	1.5	0.1	RT	0%(0)	_	_	Inf	0.02
L74I	5.6%(10)	63.5	10.6	RT	1.1%(5)	5.0	2.5	5.6	<0.01
K103N	37.1%(66)	49.6	73.3	RT	18.1%(86)	43.1	77.6	2.7	<0.01
V106A	2.2%(4)	1.3	0.1	RT	0%(0)	_	_	Inf	<0.01
**E169D**	9.6%(17)	13.5	14.4	RT	4.4%(21)	3.8	12.8	2.3	0.02
Y181C	23%(41)	41.0	19.8	RT	14.5%(69)	11.5	33.3	1.8	0.01
G190A	15.7%(28)	19.5	28.2	RT	7.8%(37)	12.7	32.4	2.2	<0.01
**Q197K**	1.7%(3)	2.1	1.9	RT	0%(0)	_	_	Inf	0.02
**T200V**	3.4%(6)	8.0	Inf	RT	0.6%(3)	5.0	Inf	5.5	0.02
**T200E**	10.7%(19)	19.0	Inf	RT	5.5%(26)	26.0	Inf	2.1	0.03
T215I	1.7%(3)	3.9	0.0	RT	0%(0)	_	_	Inf	0.02
**E224D**	1.7%(3)	1.4	1.3	RT	0%(0)	_	_	Inf	0.02
P225H	1.7%(3)	1.2	1.2	RT	0%(0)	_	_	Inf	0.02

Note:

^a^ The bold mutations were those that have not been reported to be associated with drug resistance.

^b^ P-value was computed by using a chi-square test or a Fisher's exact test.

^c^ This value was infinite.

### Covariation Mutation and Network Graph Analysis

Table 3 indicated the Jaccard similarity coefficients and conditional probabilities of the positively associated protease mutation pairs between death-associated polymorphisms and drug resistance mutations. Only a small number of mutations were found in the HIV protease, so this covariation analysis only considered mutations of the reverse transcriptase. 6 polymorphisms that were not previously identified as being associated with drug resistance per the Stanford database (T39A, K43E, S68N, Q197K, T200V and E224D) were found to have an association with drug resistance mutations. There was a correlation between T39A and three mutations (M41L, K43E, and M184V) known to confer drug resistance.

**Table 3:** Covariation pairs between death-associated polymorphisms and other mutations in the deceased group.

Mutation 1^a^	Mutation 2^b^	OR	JI^c^	p	Mutation 1	Mutation 2	OR	JI	p
**E6D**	**K70R**	6.7	0.2	0.008	Y181C	E203K	Inf	0.1	0.003
**E6D**	**S162H**	Inf	0.1	0.003	Y181C	H221Y	16.5	0.4	0.000
**E6D**	**T200V**	11.2	0.2	0.002	Y181C	K101E	16.9	0.2	0.000
**T39A**	**K43E**	47.5	0.2	0.006	Y181C	K65R	Inf	0.1	0.001
**T39A**	**M184V**	Inf	0.1	0.003	Y181C	L228R	Inf	0.3	0.000
**T39A**	**M41L**	Inf	0.1	0.002	Y181C	L74V	14.6	0.2	0.000
**K43E**	**D218E**	42.6	0.3	0.001	Y181C	M184V	4.3	0.2	0.002
**K43E**	**D67N**	10.0	0.2	0.005	Y181C	M41L	4.2	0.2	0.005
**K43E**	**E203D**	14.5	0.2	0.005	Y181C	T215F	20.8	0.1	0.001
**K43E**	**E224D**	47.5	0.2	0.006	Y181C	V108I	8.2	0.2	0.002
**K43E**	**E44A**	Inf	0.4	0.000	G190A	D237E	Inf	0.1	0.004
**K43E**	**G190S**	Inf	0.4	0.000	G190A	D67N	6.7	0.2	0.001
**K43E**	**G196E**	Inf	0.4	0.000	G190A	E203D	7.1	0.2	0.008
**K43E**	**K101E**	20.5	0.3	0.001	G190A	H221Y	7.7	0.3	0.000
**K43E**	**L210W**	Inf	0.5	0.000	G190A	K101E	7.1	0.2	0.003
**K43E**	**L74V**	23.9	0.3	0.000	G190A	K103S	Inf	0.1	0.004
**K43E**	**M184V**	Inf	0.3	0.000	G190A	K219E	22.1	0.1	0.003
**K43E**	**R211K**	14.9	0.2	0.001	G190A	M41L	4.9	0.2	0.002
**K43E**	**T215Y**	22.5	0.2	0.000	G190A	V108I	14.6	0.2	0.000
**K43E**	**V118I**	133.2	0.4	0.000	G190A	V179I	4.7	0.2	0.007
**K43E**	**V179I**	11.8	0.2	0.003	Q197K	I202V	56.3	0.3	0.005
**K43E**	**V35R**	Inf	0.3	0.002	Q197K	K219Q	125.5	0.4	0.001
**S68N**	**E203D**	41.0	0.2	0.008	Q197K	K70R	36.0	0.2	0.010
**L74I**	**D177N**	Inf	0.7	0.000	Q197K	T200E	Inf	0.2	0.001
**L74I**	**D218E**	89.1	0.3	0.002	Q197K	T215F	56.3	0.3	0.005
**L74I**	**D67S**	Inf	0.7	0.000	T200E	F227L	26.2	0.2	0.005
**L74I**	**E203D**	Inf	0.3	0.000	T200E	H221Y	6.9	0.3	0.000
**L74I**	**K103H**	Inf	0.7	0.000	T200E	K219Q	26.2	0.2	0.005
**L74I**	**K166R**	125.5	0.4	0.001	T200E	Q197K	Inf	0.2	0.001
**L74I**	**K219E**	89.1	0.3	0.002	T200E	R211G	12.7	0.3	0.000
**L74I**	**M184V**	Inf	0.1	0.003	T215I	A158S	Inf	0.7	0.000
**L74I**	**S162D**	Inf	0.7	0.000	T215I	A62V	41.0	0.2	0.008
**L74I**	**S68G**	41.0	0.2	0.008	T215I	E203R	Inf	0.7	0.000
**L74I**	**T215F**	56.3	0.3	0.005	T215I	E40R	Inf	0.7	0.000
**L74I**	**V108I**	36.0	0.2	0.010	T215I	E6K	69.2	0.3	0.003
**L74I**	**V118I**	89.1	0.3	0.002	T215I	G112S	Inf	0.7	0.000
**L74I**	**V35L**	89.1	0.3	0.002	T215I	K219H	Inf	0.7	0.000
**L74I**	**V75S**	Inf	0.7	0.000	T215I	K223E	Inf	0.7	0.000
**K103N**	**Y181C**	3.2	0.3	0.002	T215I	K46Q	Inf	0.7	0.000
**V106A**	**F227L**	67.7	0.3	0.003	T215I	K65R	89.1	0.3	0.002
**V106A**	**K70G**	Inf	0.5	0.000	T215I	S68G	41.0	0.2	0.008
**V106A**	**Q151M**	46.9	0.3	0.004	E224D	A62V	41.0	0.2	0.008
**E169D**	**A62V**	8.6	0.2	0.007	E224D	K101Q	89.1	0.3	0.002
**E169D**	**D123E**	4.8	0.2	0.010	E224D	V35R	Inf	0.7	0.000
**E169D**	**V179I**	7.4	0.2	0.002	E224D	V75T	125.5	0.4	0.001
**E169D**	**V35R**	Inf	0.1	0.010	E224D	Y188L	56.3	0.3	0.005
**E169D**	**V75T**	30.2	0.2	0.003	P225H	V106A	125.5	0.4	0.001
**Y181C**	**D218E**	Inf	0.1	0.001	P225H	V90I	41.0	0.2	0.008

Note:

^a^ Mutation 1 were death-associated polymorphisms in the deceased group.

^b^ Mutation 2 were other mutations in the deceased group.

^c^Jaccard similarity coefficient, also known as Jaccard index (JI), was used to measure the covariation among mutations.

**Fig 1:**
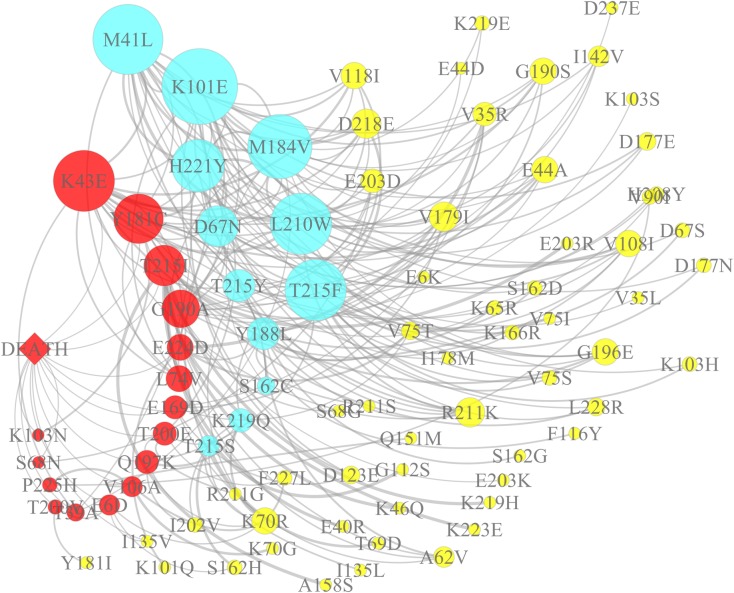
Interactive network of death-associated polymorphisms and other mutations. The network represents the global relationship among the death-associated polymorphisms and other mutations. The death-associated polymorphisms are highlighted in red, the drug resistance mutations are highlighted in green, and all other mutations are yellow. In the network, the size of the node represents the mutation frequency of that site from one amino acid to another, while the width of the line represents the strength of the influence between two mutations.

A more complex relationship between these mutations can be seen in Fig 1, which is advantageous in understanding the covariation among mutations. Additionally, once this relationship is understood, it will help to reveal the underlying mutation mechanisms. The network was stratified into three layers based on the three types of mutations (death-associated polymorphisms, drug resistance mutations, and other mutations). Death-associated polymorphisms are displayed in the left layer (some of them are drug resistance mutations, while drug resistance mutations are displayed in the middle layer and other mutations in the right layer. In the network, drug resistance mutations with higher covariation (e.g., M41L, D67N, K101E, K103N, Y181C, M184V, L210W, T215F, and T215I) were more likely to influence the other mutations. For example, M41L and D67N had 16 (D218E, E44A, G190S, G196E, H221Y, I142V, K101E, L210W, L228R, L74V, M184V, R211K, T215F, V108I, V118I, and V179I) and nine (E44A, H208Y, H221Y, K70R, L210W, M184V, M41L, R211K, and V90I) target mutations, respectively.

In our covariation analysis, four death-associated mutations (T39A, K43E, L74I, and Y181C) were significantly associated with M184V. Non-drug resistant but death-associated polymorphisms T39A may play a role in promoting drug resistance through M184V. The emergence of these accessory mutations could be related to the evolutionary pathway leading to drug resistance. In the deceased group, the covariation pairs between M184V and L74I were highly significant (p < 0.01, OR > 1). M41L was significantly associated with T215Y (p < 0.001; R > 1). L210W was significantly associated with M41L and T215Y (p < 0.001; R > 1). The rates of M41L in the deceased and survival groups were not significant. However, in the deceased group, M41L was significantly associated with the TAMs (thymidine analogue resistance mutations) D67N, L210W, and T215Y/F. This probably increased both drug resistance and adaptability. D67N was significantly associated with the other TAMs K70R, H208Y, L210W, and T215Y. V118I was significantly associated with M41L, L210W, and T215F in this cluster (p < 0.001; R > 1).

## Discussion

The increasing evidences suggest that in addition to those currently known mutations, more and more unidentified mutations may also be involved in the development of drug resistance, which contribute to therapy failure, and the development of drug resistance may be more complex than the classical one-step model of significant resistance via a single mutation so far considered [23, 24]. Some studies suggest that the various subtypes may respond differently to ARV drugs [25, 26]. The frequency and pattern of mutations conferring resistance to these drugs differ among HIV-1 subtypes and can influence the outcome [27]. HIV-1 Subtype B' plays unique roles in the genesis of the HIV-1 epidemic in China. As a result, it is particularly important to understand the mutation changes in patients infected by HIV-1 subtype B' and their effect on dug-resistance.

In this study, death-associated polymorphisms emerged after the viral failure (i.e., the inability to achieve or maintain viral suppression, or a viral load of more than 1,000 copies/mL) and were found more than one year prior to the death of the patients. As such, these polymorphisms that were not reported to be involved in drug resistance might be considered drug resistance mutations or compensatory mutations. In this study, about 36 (5.5%) samples sequenced in the beginning, and they might not under enough selective therapy pressure, but no significant mutations were found in these samples.

Among 20 significant death-associated polymorphisms, 13 (65%) polymorphisms (E6D, Q18H, E35D, S37N, T39A, K43E, S68N, I93L, E169D, Q197K, T200V, T200E and E224D) had not been previously reported to be drug resistance mutations, but they were strongly associated with death. 7 (35%) mutations (E6D, E35D, S37N, I93L, E169D, T200V, and T200E) were considered to be potential drug resistance mutations in this study. 6 polymorphic mutations (Q18H, K43E, S68N, Q197K, T215I and E224D) were presented in strains isolated from the deceased patients, while they were completely absent in the strains isolated from the living patients, and they were all positive Darwin selection mutations (*Ka*/*Ks* > 1, LOD > 2), but LOD≤2. *Ka*/*Ks* calculations successfully identified the mutations that were associated with drug resistance and AIDS-related death. However, many researchers had reported that drug resistance mutations displayed positive selection (*Ka*/*Ks* > 1) with LOD scores greater than 2 [12, 13, 28]. Therefore, these polymorphisms differed from drug resistance mutations in the traditional sense. Some studies had shown that accessory mutations at positions 39 (T39A), 43 (K43E) were more frequent in viral isolates from patients failing therapy than in naive individuals, and those mutations were previously identified as accessory mutations associated with the accumulation of TAMs (thymidine analogue resistance mutations) and with high levels of resistance to nucleoside analogues[23, 28–30]. These polymorphisms require further clinical data and related literature to demonstrate the nature of this difference.

This study also found these polymorphisms had complex network relationships with previously reported drug resistance mutations. Stratified networks were utilized to display the mutation covariation and had proved useful in studying mutation variation during ART [12, 28, 31]. It had been reported that HIV can employ various combinations of mutations to resist drug treatments[32]. To determine mutational interactions between the newly identified and drug resistance mutations in RT of HIV-1 subtype B' strains, we used the Jaccard similarity coefficient and network graph to investigate the correlated mutations in the deceased group, and found that these newly identified mutations were likely to be related to drug resistance. Network showed that most mutations were connected together as a component; mutations of high frequency were more likely to influence the other mutations (Fig 1). The relationship among mutations in the networks could give clues to the combinatorial mutation patterns responsible for drug resistance within the network. For example, T39A and K43E had three (M41L, K43E, and M184V) and seven (D67N, L74V, K101E, L210W, M184V, T215Y, and E224D) target drug resistance mutations, respectively. The other two non-drug-resistance mutations (E6D and E169D) also had indirect relationship with drug resistance mutations (e.g., E6D—K70R—Q197K; E169D—V179I—G190A), indicating a possible association between these mutations and drug resistance. Several studies had reported that HIV-1 replication efficiency might correlate with disease progression [33–35]. Understanding these polymorphisms could help to assess the most at-risk populations to avoid undermining current treatment regimens, achieve the greatest impact for the most people, and ensure sustainability. Thus, further *in vitro* experiments are required to confirm whether the death-associated mutations are drug resistance mutations or compensatory mutations, as well as elucidate the role of these mutations in the development of drug resistance.

This study also had several limitations. 1) The sample size was not large enough and did not include all kinds of patients in China; and 2) covariance analysis of related protein sequences was known to be a few problematic [36]. Jaccard similarity coefficient treated all substitutions of amino acids equally, ignoring physicochemical preferences. It may be worth considering different essential covariance measures for further analysis, and conclusions should be made with caution. 3) Not only resistance mutations or polymorphisms, but other biases could interfere with the progression to death, such as age, physical condition, combination antiretroviral regimens, CD4 + cell count baseline, pathologies presented at the beginning of the follow-up and so on. Therefore, a more detailed analysis of stratification will be in further consideration; 4) A small number of mutations were found in HIV protease, so this method was not able to analyze the correlation between them.

## Supporting Information

S1 File Relevant data underlying the findings described in manuscript. (SAS7BDAT)

S2 File Sequence of this research. (FASTA)

## References

[1] NCAIDS, NCSTD, and China CDC. Update on the AIDS/STD epidemic in China and main response in control and prevention in December, 2015. Chin J AIDS STD 2016.

[2] Zhang F, Haberer JE, Wang Y, Zhao Y, Ma Y, Zhao D et al The Chinese free antiretroviral treatment program: challenges and responses. Aids 2007 12;21 Suppl 8:S143–8. 18172383 10.1097/01.aids.0000304710.10036.2b

[3] Wang J, Wang Z, Liu J, Yue Y, Yang S, Huang H et al Efficacy and HIV drug resistance profile of second-line ART among patients having received long-term first-line regimens in rural China. Scientific reports 2015;5:14823 10.1038/srep14823 26445885 PMC4597210

[4] Ma Y, Zhao D, Yu L, Bulterys M, Robinson ML, Zhao Y et al. Predictors of virologic failure in HIV-1-infected adults receiving first-line antiretroviral therapy in 8 provinces in China. Clinical infectious diseases: an official publication of the Infectious Diseases Society of America 2010 1 15;50(2):264–71. 20017637 10.1086/649215 PMC2805417

[5] Zhang F, Dou Z, Ma Y, Zhang Y, Zhao Y, Zhao D et al Effect of earlier initiation of antiretroviral treatment and increased treatment coverage on HIV-related mortality in China: a national observational cohort study. The Lancet Infectious diseases 2011 7;11(7):516–24. 10.1016/S1473-3099(11)70097-4 21600849

[6] Mahalingam B, Boross P, Wang YF, Louis JM, Fischer CC, Tozser J et al Combining mutations in HIV-1 protease to understand mechanisms of resistance. Proteins 2002 7 1;48(1):107–16. 10.1002/prot.10140 12012342

[7] Ohtaka H, Schon A, Freire E. Multidrug resistance to HIV-1 protease inhibition requires cooperative coupling between distal mutations. Biochemistry 2003 11 25;42(46):13659–66. 10.1021/bi0350405 14622012

[8] Rousseau MN, Vergne L, Montes B, Peeters M, Reynes J, Delaporte E et al Patterns of resistance mutations to antiretroviral drugs in extensively treated HIV-1-infected patients with failure of highly active antiretroviral therapy. Journal of acquired immune deficiency syndromes 2001 1 1;26(1):36–43. 11176267 10.1097/00126334-200101010-00005

[9] Barbour JD, Wrin T, Grant RM, Martin JN, Segal MR, Petropoulos CJ et al Evolution of phenotypic drug susceptibility and viral replication capacity during long-term virologic failure of protease inhibitor therapy in human immunodeficiency virus-infected adults. Journal of virology 2002 11;76(21):11104–12. 10.1128/JVI.76.21.11104-11112.2002 12368352 PMC136622

[10] Sturmer M, Staszewski S, Doerr HW, Larder B, Bloor S, Hertogs K. Correlation of phenotypic zidovudine resistance with mutational patterns in the reverse transcriptase of human immunodeficiency virus type 1: interpretation of established mutations and characterization of new polymorphisms at codons 208, 211, and 214. Antimicrobial agents and chemotherapy 2003 1;47(1):54–61. 10.1128/AAC.47.1.54-61.2003 12499169 PMC149009

[11] Rhee SY, Fessel WJ, Zolopa AR, Hurley L, Liu T, Taylor J et al HIV-1 Protease and reverse-transcriptase mutations: correlations with antiretroviral therapy in subtype B isolates and implications for drug-resistance surveillance. The Journal of infectious diseases 2005 8 1;192(3):456–65. 10.1086/431601 15995959 PMC2597526

[12] Li Z, Huang Y, Ouyang Y, Xing H, Liao L, Jiang S et al Mutation covariation of HIV-1 CRF07_BC reverse transcriptase during antiretroviral therapy. The Journal of antimicrobial chemotherapy 2013 11;68(11):2521–4. 10.1093/jac/dkt228 23788482

[13] Chen L, Perlina A, Lee CJ. Positive selection detection in 40,000 human immunodeficiency virus (HIV) type 1 sequences automatically identifies drug resistance and positive fitness mutations in HIV protease and reverse transcriptase. Journal of virology 2004 4;78(7):3722–32. 10.1128/JVI.78.7.3722-3732.2004 15016892 PMC371046

[14] Rhee SY, Liu TF, Holmes SP, Shafer RW. HIV-1 subtype B protease and reverse transcriptase amino acid covariation. PLoS computational biology 2007 5;3(5):e87 10.1371/journal.pcbi.0030087 17500586 PMC1866358

[15] Zhao F, Wang Z, Li WJ. Human immunodeficiency virus type 1 subtypes prevalence in central China. Yonsei medical journal 2009 10 31;50(5):644–9. 10.3349/ymj.2009.50.5.644 19881967 PMC2768238

[16] Liao L, Xing H, Su B, Wang Z, Ruan Y, Wang X et al Impact of HIV drug resistance on virologic and immunologic failure and mortality in a cohort of patients on antiretroviral therapy in China. Aids 2013 7 17;27(11):1815–24. 10.1097/QAD.0b013e3283611931 23803794 PMC3694318

[17] Zhang J, Shen ZY, Li Z, Liang SJ, He C, Liang FX et al Genetic Characteristics of CRF01_AE Among Newly Diagnosed HIV-1-Infected 16- to 25-Year Olds in 3 Geographic Regions of Guangxi, China. Medicine 2015 5;94(21):e894 10.1097/MD.0000000000000894 26020400 PMC4616409

[18] Guindon S, Dufayard JF, Lefort V, Anisimova M, Hordijk W, Gascuel O. New algorithms and methods to estimate maximum-likelihood phylogenies: assessing the performance of PhyML 3.0. Systematic biology 2010 5;59(3):307–21. 10.1093/sysbio/syq010 20525638

[19] Zhang X, Li S, Li X, Li X, Xu J, Li D et al Characterization of HIV-1 subtypes and viral antiretroviral drug resistance in men who have sex with men in Beijing, China. Aids 2007 12;21 Suppl 8:S59–65. 10.1097/01.aids.0000304698.47261.b1 18172393

[20] Yang Z. Inference of selection from multiple species alignments. Current opinion in genetics & development 2002 12;12(6):688–94. 12433583 10.1016/s0959-437x(02)00348-9

[21] Li WH. Unbiased estimation of the rates of synonymous and nonsynonymous substitution. Journal of molecular evolution 1993 1;36(1):96–9. 8433381 10.1007/BF02407308

[22] Shannon P, Markiel A, Ozier O, Baliga NS, Wang JT, Ramage D et al Cytoscape: a software environment for integrated models of biomolecular interaction networks. Genome research 2003 11;13(11):2498–504. 10.1101/gr.1239303 14597658 PMC403769

[23] Ceccherini-Silberstein F, Svicher V, Sing T, Artese A, Santoro MM, Forbici F et al Characterization and structural analysis of novel mutations in human immunodeficiency virus type 1 reverse transcriptase involved in the regulation of resistance to nonnucleoside inhibitors. Journal of virology 2007 10;81(20):11507–19. 10.1128/JVI.00303-07 17686836 PMC2045529

[24] Bacheler LT, Anton ED, Kudish P, Baker D, Bunville J, Krakowski K et al Human immunodeficiency virus type 1 mutations selected in patients failing efavirenz combination therapy. Antimicrobial agents and chemotherapy 2000 9;44(9):2475–84. 10952598 10.1128/aac.44.9.2475-2484.2000 PMC90088

[25] Pelemans H, Esnouf R, Dunkler A, Parniak MA, Vandamme AM, Karlsson A et al Characteristics of the Pro225His mutation in human immunodeficiency virus type 1 (HIV-1) reverse transcriptase that appears under selective pressure of dose-escalating quinoxaline treatment of HIV-1. Journal of virology 1997 11;71(11):8195–203. 9343170 10.1128/jvi.71.11.8195-8203.1997 PMC192276

[26] Singh K, Flores JA, Kirby KA, Neogi U, Sonnerborg A, Hachiya A et al Drug resistance in non-B subtype HIV-1: impact of HIV-1 reverse transcriptase inhibitors. Viruses 2014 9;6(9):3535–62. 10.3390/v6093535 25254383 PMC4189038

[27] Richman D, Shih CK, Lowy I, Rose J, Prodanovich P, Goff S et al Human immunodeficiency virus type 1 mutants resistant to nonnucleoside inhibitors of reverse transcriptase arise in tissue culture. Proceedings of the National Academy of Sciences of the United States of America 1991 12 15;88(24):11241–5. 1722324 10.1073/pnas.88.24.11241 PMC53110

[28] Huang Y, Li Z, Xing H, Jiao Y, Ouyang Y, Liao L et al Identification of the critical sites of NNRTI-resistance in reverse transcriptase of HIV-1 CRF_BC strains. PloS one 2014;9(4):e93804 10.1371/journal.pone.0093804 24743727 PMC3990534

[29] Pan C, Kim J, Chen L, Wang Q, Lee C. The HIV positive selection mutation database. Nucleic acids research 2007 1;35(Database issue):D371–5. 10.1093/nar/gkl855 17108357 PMC1669717

[30] Zolopa AR, Shafer RW, Warford A, Montoya JG, Hsu P, Katzenstein D et al HIV-1 genotypic resistance patterns predict response to saquinavir-ritonavir therapy in patients in whom previous protease inhibitor therapy had failed. Annals of internal medicine 1999 12 7;131(11):813–21. 10610625 10.7326/0003-4819-131-11-199912070-00003 PMC2606144

[31] Liu Y, Eyal E, Bahar I. Analysis of correlated mutations in HIV-1 protease using spectral clustering. Bioinformatics 2008 5 15;24(10):1243–50. 10.1093/bioinformatics/btn110 18375964 PMC2373918

[32] Zhang J, Hou T, Wang W, Liu JS. Detecting and understanding combinatorial mutation patterns responsible for HIV drug resistance. Proceedings of the National Academy of Sciences of the United States of America 2010 1 26;107(4):1321–6. 10.1073/pnas.0907304107 20080674 PMC2824344

[33] Deacon NJ, Tsykin A, Solomon A, Smith K, Ludford-Menting M, Hooker DJ et al Genomic structure of an attenuated quasi species of HIV-1 from a blood transfusion donor and recipients. Science 1995 11 10;270(5238):988–91. 7481804 10.1126/science.270.5238.988

[34] Kirchhoff F, Greenough TC, Brettler DB, Sullivan JL, Desrosiers RC. Brief report: absence of intact nef sequences in a long-term survivor with nonprogressive HIV-1 infection. The New England journal of medicine 1995 1 26;332(4):228–32. 10.1056/NEJM199501263320405 7808489

[35] Learmont J, Tindall B, Evans L, Cunningham A, Cunningham P, Wells J et al Long-term symptomless HIV-1 infection in recipients of blood products from a single donor. Lancet 1992 10 10;340(8824):863–7. 1357294 10.1016/0140-6736(92)93281-q

[36] Fodor AA, Aldrich RW. Influence of conservation on calculations of amino acid covariance in multiple sequence alignments. Proteins 2004 8 1;56(2):211–21. 10.1002/prot.20098 15211506

